# The histone variant macroH2A confers functional robustness to the intestinal stem cell compartment

**DOI:** 10.1371/journal.pone.0185196

**Published:** 2017-09-21

**Authors:** Ryan James Cedeno, Angela Nakauka-Ddamba, Maryam Yousefi, Stephanie Sterling, Nicolae Adrian Leu, Ning Li, John R. Pehrson, Christopher Joachim Lengner

**Affiliations:** 1 Department of Biomedical Sciences, University of Pennsylvania School of Veterinary Medicine, Philadelphia, Pennsylvania, United States of America; 2 Cell and Molecular Biology Graduate Program, Perelman School of Medicine at the University of Pennsylvania, Philadelphia, United States of America; 3 Center for Animal Transgenesis, University of Pennsylvania School of Veterinary Medicine, Philadelphia, Pennsylvania, United States of America; 4 Center for Molecular Studies in Digestive and Liver Disease, University of Pennsylvania, Philadelphia, Pennsylvania, United States of America; 5 Institute for Regenerative Medicine, University of Pennsylvania, Philadelphia, Pennsylvania, United States of America; National Cancer Institute, UNITED STATES

## Abstract

A stem cell’s epigenome directs cell fate during development, homeostasis, and regeneration. Epigenetic dysregulation can lead to inappropriate cell fate decisions, aberrant cell function, and even cancer. The histone variant macroH2A has been shown to influence gene expression, guide cell fate, and safeguard against genotoxic stress. Interestingly, mice lacking functional macroH2A histones (hereafter referred to as macroH2A DKO) are viable and fertile; yet suffer from increased perinatal death and reduced weight and size compared to wildtype (WT). Here, we ask whether the ostensible reduced vigor of macroH2A DKO mice extends to intestinal stem cell (ISC) function during homeostasis, regeneration, and oncogenesis. *Lgr5-eGFP-IRES-CreERT2* or *Hopx-CreERT2*::*Rosa26-LSL-tdTomato* ISC reporter mice or the *C57BL/6J-Apc*^*min*^*/J* murine intestinal adenoma model were bred into a macroH2A DKO or strain-matched WT background and assessed for ISC functionality, regeneration and tumorigenesis. High-dose (12Gy) whole-body γ-irradiation was used as an injury model. We show that macroH2A is dispensable for intestinal homeostasis and macroH2A DKO mice have similar numbers of active crypt-base columnar ISCs (CBCs). MacroH2A DKO intestine exhibits impaired regeneration following injury, despite having significantly more putative reserve ISCs. DKO reserve ISCs disproportionately undergo apoptosis compared to WT after DNA damage infliction. Interestingly, a macroH2A DKO background does not significantly increase tumorigenesis in the *Apc*^*min*^ model of intestinal adenoma. We conclude that macroH2A influences reserve ISC number and function during homeostasis and regeneration. These data suggest macroH2A enhances reserve ISC survival after DNA damage and thus confers functional robustness to the intestinal epithelium.

## Introduction

The intestinal epithelium is the most highly proliferative mammalian tissue. Its rapid turnover and tremendous regenerative capacity following injury necessitate a robust and highly organized ISC compartment. ISCs are located within the intestinal crypt where they self-renew and produce progenitors, which in turn proliferate and terminally differentiate along the crypt-villus axis prior to being shed into the lumen. To accommodate this rapid turnover and respond to environmental cues, the intestine is served by at least two functionally distinct ISC populations, including the fast-cycling CBCs and slow-cycling reserve ISCs.[1]

CBCs are marked by expression of Wnt-responsive G-protein coupled receptor Lgr5, are driven to actively proliferate by canonical Wnt pathway activity, strongly contribute to intestinal homeostasis[2, 3] and are ablated by γ-irradiation.[3–7] In contrast, reserve ISCs are rare, largely quiescent, radioresistant, and can be marked by *CreER* reporter genes inserted into the *Bmi1*, *or Hopx* loci, as well as by transgenes driven by the *mTert* and *Lrig1* promoters.[4, 8–14] Following DNA damage and CBC loss, reserve ISCs awaken en masse and play a critical role in epithelial regeneration–in part by producing CBCs.[4, 15, 16] Epigenetic mechanisms governing the identities of these two classes of ISCs have not been investigated.

An underappreciated facet of epigenetic control is the substitution of canonical core histones for structural variants. One such variant–macroH2A[17], is highly conserved[18, 19] and is implicated in reinforcing cell identity *in vitro*.[20–23] Structurally, macroH2A consists of a histone domain, a linker, and a large globular non-histone domain that renders macroH2A about three times the size of canonical core histone H2A.[17] MacroH2A is enriched at both facultative and constitutive heterochromatin including the Xi,[24–29] senescence-associated heterochromatin foci,[30, 31] lamin-associated domains[32] and other transcriptionally silent chromatin.[29, 33, 34] MacroH2A has been implicated in transcriptional silencing via mechanisms including blocking recruitment of the SWI/SNF nucleosome remodeling complex,[35, 36] repressing p300 and Gal-VP16-driven RNA pol II transcriptional initiation,[37] and modulating Parp-1.[38, 39] Interestingly, some active chromatin domains also contain macroH2A,[34] but at least a subset of these sites undergo dynamic macroH2A incorporation and turnover (rather than long-term, stable deposition) and remain transcriptionally accessible.[40]

In mammals, macroH2A exists as 3 isoforms encoded by 2 genes–*H2afy* encodes splice variants macroH2A1.1 and macroH2A1.2, and *H2afy2* encodes macroH2A2.[20, 41, 42] MacroH2A1.1 facilitates chromatin remodeling by binding Parp-1 and ADP-ribosylated chromatin, a property the other macroH2As lack.[43, 44] Global macroH2A chromatin content increases during development,[20, 45, 46] and macroH2A removal has been described as an epigenetic bottleneck to induced pluripotency.[46–48] Interestingly, macroH2A chromatin content also increases with tissue age,[31] coincident with the known loss of stem cell vigor in aging. Similarly, macroH2A overexpression limits stem cell self-renewal *in vitro*.[49] Interestingly, germline macroH2A DKO mice are viable and fertile during homeostasis, yet are peculiarly less robust than WT as evidenced by increased perinatal death and reduced body weight and size throughout life compared to WT.[19] In line with a role for macroH2A in conferring robustness, macroH2A has been shown in cell lines to provide resistance against varied forms of genotoxic stress.[38, 50–53] These *in vitro* studies suggest that macroH2A, while perhaps dispensable during homeostasis, may similarly provide cells and even tissues at large with stress resistance *in vivo*.

Here, we show that macroH2A DKO mice have normal intestinal epithelial function during homeostasis. However, macroH2A DKO intestine exhibits reduced regeneration following γ-irradiation injury. Seemingly paradoxically, macroH2A DKO intestine contains markedly more reserve ISCs, but these ISCs are significantly more radiosensitive than WT counterparts. Lastly, we observe no elevated levels of intestinal adenoma formation in the *Apc*^*min/+*^ intestinal transformation model in a macroH2A DKO background, corroborating the observed lack of spontaneous tumorigenesis in macroH2A DKO mice[19] despite evidence that suggests macroH2As may have tumor suppressive properties.[54–58] Our study demonstrates that the histone variant macroH2A, despite being dispensable during intestinal homeostasis and of limited overall influence on intestinal adenoma growth, nevertheless bestows the ISC compartment with functional robustness, specifically by providing resistance to genotoxic stress.

## Materials and methods

### Mouse strains

All mouse experiments were approved by and performed under the purview of the University of Pennsylvania’s Institutional Animal Care and Use Committee (IACUC) under protocol 803415 granted to Dr. Lengner. *Lgr5-eGFP-IRES-CreERT2* (JAX strain 008875) mice were acquired from The Jackson Laboratory. *Hopx-CreERT2* (JAX strain 017606) mice were a kind gift from Dr. Jon Epstein, and macroH2A DKO (JAX strain 025481) were kindly provided by Dr. John Pehrson. MacroH2A DKO and strain-matched 129S1/SvIm mice were crossed with *Lgr5-eGFP-IRES-CreERT2* or *Hopx-CreERT2*::*Rosa26-LSL-tdTomato* mice. *C57BL/6J-Apc*^*min*^*/J* mice were obtained from Jackson Laboratory (JAX strain 002020) and bred into a macroH2A DKO background in parallel with WT 129S1/SvIm mice. All mice were sacrificed for analysis at 2 months of age unless indicated otherwise. Mice were humanely sacrificed by CO_2_ asphyxia followed by cervical dislocation as outlined by approved University of Pennsylvania IACUC protocols.

### Histology

Histology was performed at the Molecular Pathology & Imaging Core (MPIC) of the Penn Center for Molecular Studies in Digestive and Liver Diseases. In brief, mouse small intestines were washed with DPBS and fixed overnight at 4°C in Zinc formalin (Polysciences Inc.). Following sectioning and tissue deparaffanization, antigen retrieval was performed with 10mM Tris base (pH 9.0) buffer using a pressure cooker.

For immunohistochemistry, sections were quenched of endogenous peroxidases by 3% H2O2, and sequentially blocked with Avidin D, biotin, and protein blocking reagents. Primary antibody incubation was conducted at 4°C overnight. Secondary biotinylated antibody was added at a dilution of 1:200, and incubated 2 hours at room temperature. Finally, sections were stained according to the ABC peroxidase protocol (Vector Laboratories) and counterstained with haematoxylin. Images were taken using an inverted Leica DM IRB microscope and analysis was performed using iVision software.

For immunofluorescence, sections were blocked with protein blocking reagent and incubated with primary antibody overnight at 4°C. Sections were washed in PBS and stained with fluorescent secondary antibodies (Jackson Laboratories) and counterstained with DAPI (Vector Laboratories). For immunofluorescence using mouse primary antibodies, a mouse-on-mouse (MOM) kit was employed (Vector Laboratories). Images were taken using a Nikon E600 microscope and fluorescent channel overlay and analysis was performed using iVision software. Specific primary antibodies and dilutions used were as follows: macroH2A1 (Abcam Ab37264, 1:200), macroH2A1.1 (CST #12455, 1:200), tdTomato (ClonTech 632392, 1:200), Ki67 (Abcam Ab15580, 1:200), Lysozyme C (Santa Cruz sc-27958, 1:200) ChgA (Abcam Ab15160, 1:1000), GFP (Abcam Ab6673, 1:200), cleaved caspase-3 (CST #9661) and γ-H2AX (CST #9718, 1:200).

### Isolation of intestinal epithelial cells

Mice were sacrificed and small intestine was dissected and cut open longitudinally. Villi were then scraped off using a microscope slide cover slip. Remaining tissue was then incubated with 5mM EDTA in HBSS for 30 min at 4°C to loosen crypts, and then manually pipetted up and down for mechanical dislodgement. Crypts were subsequently digested to single-cells with 0.66mg/ml Dispase (BD Biosciences).

### Flow cytometry

Flow cytometry was performed on a BD LSR Fortessa cytometer (BD Biosciences). Single cells were selected by FSC height vs. FSC width and SSC height vs. SSC width plots. For *Hopx-CreERT2*::*Rosa26-LSL-tdTomato* mice, mice were injected with 2mg tamoxifen 18h prior to sacrifice and *tdTomato*^*+*^ cells were determined via a threshold established by an injected *Hopx-WT*::*Rosa26-LSL-tdTomato* negative control. For *Lgr5-eGFP-IRES-CreERT2* mice, eGFP+ threshold was established by an *Lgr5* WT mouse. All analysis was performed using FlowJo software.

### Irradiation & regeneration, post-IR lineage tracing and apoptosis assays

For post-irradiation regeneration assessment, mice were treated with 12 Gy whole-body γ-irradiation and sacrificed 72h later at which point intestines were harvested and fixed overnight at 4°C in 4% paraformaldehyde, and processed for histology by the MPIC. Tissue sections were stained for proliferation marker Ki67. Ki67^+^ crypts per 500μm were quantitated in each section.

For post-IR lineage tracing, macroH2A WT or DKO *Hopx-CreERT2*::*Rosa26-LSL-tdTomato* mice were injected with 2mg tamoxifen 48h and 24h prior to 12 Gy whole-body γ-irradiation, and 72h later they were sacrificed. Tissues were subsequently sectioned and stained for tdTomato using the MOM immunofluorescence kit (Vector Laboratories), and tdTomato^+^ crypts were scored per 500μm.

For cleaved caspase-3 (CC3) flow cytometry, macroH2A WT or DKO *Hopx-CreERT2*::*Rosa26-LSL-tdTomato* mice were injected with 2mg tamoxifen 24h prior to 12 Gy whole-body γ-irradiation, and sacrificed 1 day later. Single crypt epithelial cells were isolated and stained with fixable viability dye (FVD) (eBioscience 65-0865-14) before BD Cytofix/Cytoperm fixation (554714) for 20 minutes at 4°C. Cells were then washed with BD Perm/Wash buffer before incubation with Pacific Blue-conjugated cleaved caspase-3 antibody (CST #8788S, 1:50) for 1 hour at 4°C. Hopx-tdTomato/CC3 double positive, FVD negative cells were then analyzed by flow cytometry.

### *In vitro* organoid formation assay

Organoid culture was performed according to a published protocol.[59] Crypt culture media consisted of Advanced DMEM/F12 supplemented with 1x B27 and N2 supplements (Invitrogen), 50 μM N-Acetylcysteine (Sigma-Aldrich), 50 ng ml^-1^ mouse EGF (Invitrogen), 1ug μL^-1^ R-Spondin (Wistar institute), 1ug μL^-1^ Noggin (Peprotech), and 3 μM GSK inhibitor CHIR99021 (Stemgent). After 7 days, intestinal organoids were qualitatively and quantitatively assessed. Organoid images were taken on a Nikon E600 microscope.

### EdU incorporation assay

*Hopx-Cre-ERT2*::*Rosa26-LSL-tdTomato* mice were injected with 2mg of tamoxifen 18 hours prior to sacrifice, and then injected with 0.3mg of 5-EdU (Thermo Fisher) per 10g of body weight 2 hours prior to sacrifice. *Lgr5-eGFP-IRES-CreERT2* mice were injected with EdU 2 hours prior to sacrifice. Crypt epithelial cells were fixed and stained for EdU according to Click-iT® EdU Alexa Fluor® 647 protocol (Thermo Fisher). DNA was counterstained with DAPI. Flow cytometric analysis was performed as stated above on populations of *tdTomato*^*+*^ or *GFP*^*+*^ cells, comparing Alexa fluor 647 fluorescence to DNA content (DAPI).

### Colorectal cancer cell proliferation (MTT) assay

RKO (ATCC stock number CRL-2577) or HCT116 (ATCC stock number CCL-247) cells were seeded in 6-well plates at 50,000 cells/well and cultured in DMEM with 10% FBS, 1% sodium pyruvate, and 1% L-glutamine 24 hours before siRNA transfection. The lipofectamine RNAiMax reagent (Invitrogen) was employed per manufacturer’s instruction. Cell proliferation was assessed using Cell Proliferation kit I protocol (Roche). Absorbance of MTT assay was measured at 570 nm. The Stealth RNAis^TM^ (Thermo Fisher) employed were siLuciferase control (Thermo Fisher 12935146), siH2AFY (Thermo Fisher HSS114259) and the macroH2A1 isoform-specific siRNAs used were of the following sequences:

siMacroH2A1.1: CACUGACUUCUACAUCGGUGGUGAA
siMacroH2A1.2: AGGCCAUAAUCAAUCCUACCAAUGC

### Apc^min^ tumorogenesis assay

MacroH2A WT or DKO; *C57BL/6J-Apc*^*min*^*/J* mice were fed a high fat / low protein diet (Research Diets, D12079B) beginning at 2 months of age, and sacrificed after 3 months on the diet to assess adenoma formation histologically. During experiments, mouse weight and health was assessed weekly, and any mice experiencing significant weight loss or apparent distress were immediately euthanized. The maximum tumor size observed in the small intestine was 3.51mm in diameter as gauged histologically.

## Results

### MacroH2A expression within the intestinal epithelium

**Fig 1:**
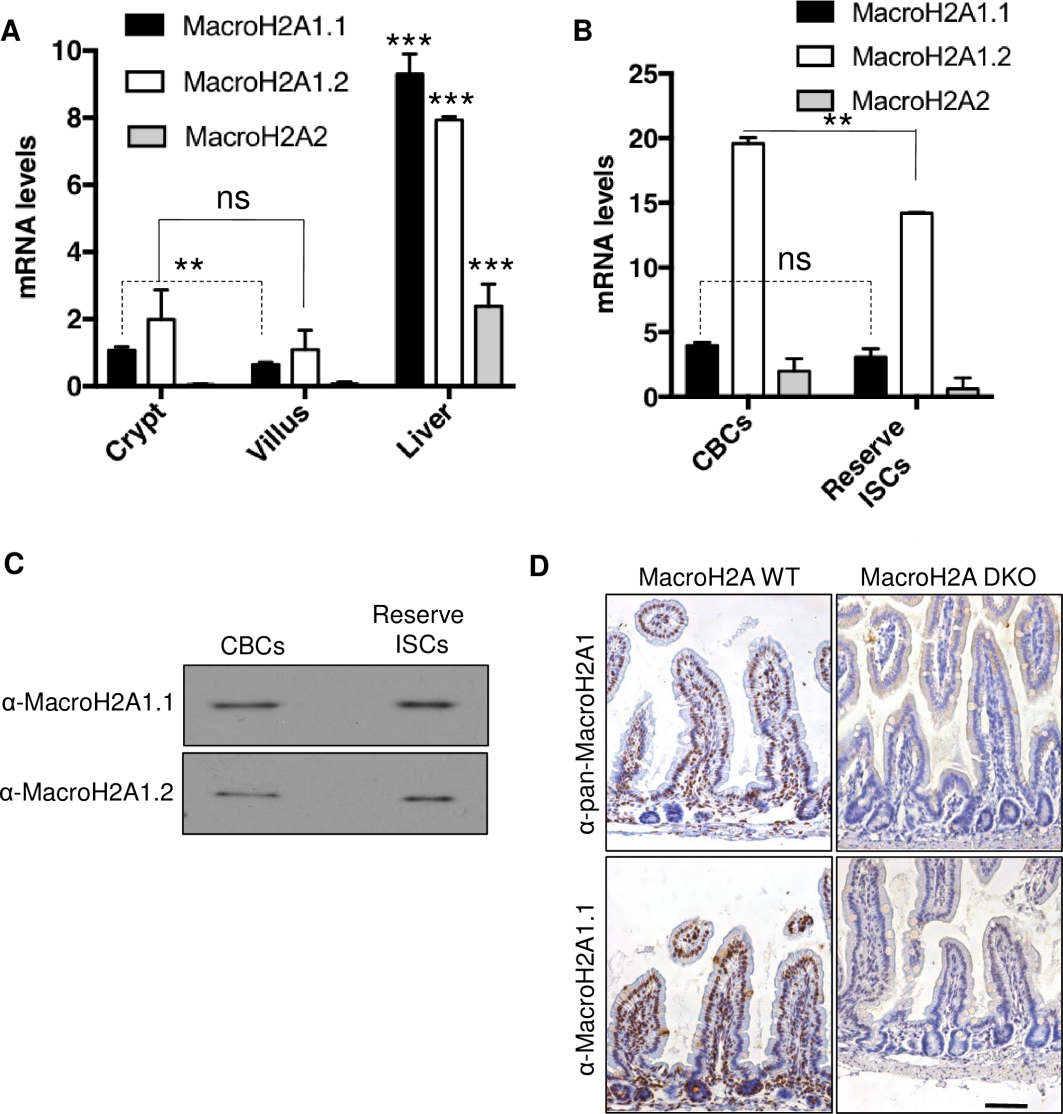
MacroH2A expression within the intestinal epithelium. (A) Analysis of intestinal jejunum crypt or villus tissue fractions for macroH2A variant mRNA levels compared to mouse liver. ΔΔCT method, values normalized to *Actb*, N = 3 per condition, mean ± SD. (B) MacroH2A isoform mRNA level analysis within Lgr5-eGFP^high^ CBCs or Hopx-tdTomato^+^ reserve ISCs FACS-purified from *Lgr5-eGFP-IRES-CreERT2* or *Hopx-CreERT2 Rosa26R-LSL-tdTomato* mice. ΔΔCT method, values normalized to *Actb*, N = 3 per condition, mean ± SD. (C) Western blot showing macroH2A1 isoform protein level within FACS-purified populations of CBCs (again, Lgr5-eGFP^high^ from *Lgr5-eGFP-IRES-CreERT2* mice) or reserve ISCs (Hopx-tdTomato^+^ from *Hopx-CreERT2 Rosa26R-LSL-tdTomato* mice). Entire protein lysate from 30,000 CBCs or 20,000 reserve ISCs loaded into each well of gel corresponding to indicated samples on blot. (D) Immunohistochemical straining of pan-macroH2A1 or macroH2A1.1 in macroH2A WT or macroH2A DKO proximal small intestine. 10x objective. Scale bars = 100μm. **p<0.005, ***p<0.0005, ns = not significant, Student’s *t*-test.

We first sought to characterize the expression of macroH2A isoforms within the intestinal epithelium. Compared to liver, a tissue known to be rich for macroH2A,[20, 41] intestinal macroH2A RNA content was at least 4-fold lower (Fig 1A). Nevertheless, *H2AFY* splice variants macroH2A1.1 and macroH2A1.2 were robustly expressed within the crypt and villus (Fig 1A). In contrast, *H2AFY2* –which encodes macroH2A2 –was not appreciably present within the small intestine (Fig 1A). Of note, the PAR-binding macroH2A1.1 was slightly enriched within the crypt versus villus (Fig 1A). Next, we FACS-purified CBCs and reserve ISCs by using the *Lgr5-eGFP-IRES-CreER[3]* and *Hopx-CreERT2*::*Rosa26-LSL-tdTomato* reporter strains respectively.[9] We use *Hopx-CreERT2* to mark reserve ISCs as we and others have shown this population to be molecularly and functionally overlapping with other reserve ISC markers including *Bmi1-CreER* and *mTert-CreER*, and single cell expression profiles indicate that the *Hopx-CreERT2* population is more homogenous that the commonly used *Bmi1-CreER* marker.[4, 8–10, 12, 13] Interestingly, the non PAR-binding macroH2A1.2 was slightly but significantly enriched within CBCs compared to reserve ISCs (Fig 1B). Further, both macroH2A1 isoforms were readily detectable at the protein level in FACS-purified ISCs (Fig 1C), and macroH2A1.1 and/or macroH2A1.2 protein was observed within most cells along the crypt-villus axis (Fig 1D). These data together delineate macroH2A expression within the intestinal epithelium and highlight the presence of at least the macroH2A1 isoforms within the tissue and ISC populations.

### MacroH2A DKO intestine during homeostasis

**Fig 2:**
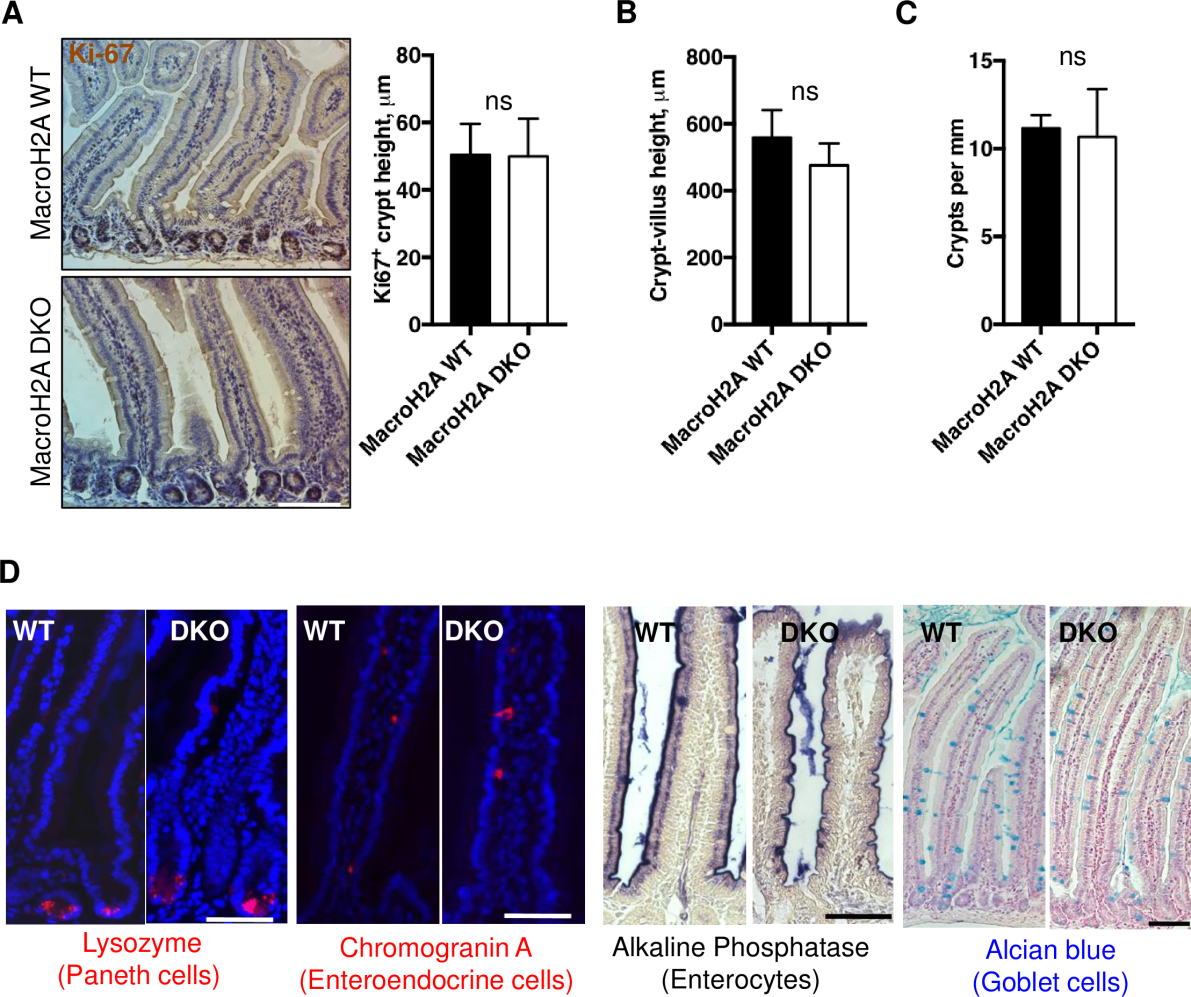
MacroH2A DKO intestine during homeostasis. (A) Left: Representative Ki67 immunohistochemistry of macroH2A WT and DKO proximal jejunum. 10x objective. Right: Average Ki67^+^ crypt height in macroH2A WT vs. DKO proximal jejunum. N = 3 mice per condition, medians, quartiles and ranges of values shown. (B) Average height in microns of crypt-villus axis (distance from base of crypt to tip of villus) of macroH2A WT vs. DKO proximal jejunum (C) Average number of crypts per mm of macroH2A WT vs. DKO proximal jejunum. (D) Representative immunofluorescence and immunohistochemical images of jejunum of macroH2A WT or DKO mice stained for lysozyme (Paneth cells), chromogranin A (enteroendocrine cells), alkaline phosphatase (enterocytes), or alcian blue (goblet cells). Immunofluorescence counterstained with DAPI (blue). Lysozyme, chromogranin A and alkaline phosphatase: 20x objective, alcian blue: 10x objective. Scale bars = 100μm. ns = not significant, Student’s *t*-test.

Next, we examined macroH2A DKO intestinal epithelia under steady-state conditions compared to WT. No gross architectural abnormalities were observed within the proximal or distal small intestine of DKO versus WT mice (Fig 2A). The Ki67^+^ crypt height and height from crypt base to villus tip in both DKO and WT intestine was comparable, (Fig 2A and 2B), as were the total number of intestinal crypts per millimeter of epithelium (Fig 2C). Both DKO and WT intestine had comparable placement and numbers of Paneth, enterocyte, enteroendocrine, and goblet cells (Fig 2D, S1 Fig). These results suggest that the intestinal epithelium does not require macroH2A histones for homeostatic maintenance.

### Total ISC activity and CBC frequency in macroH2A DKO intestine

**Fig 3:**
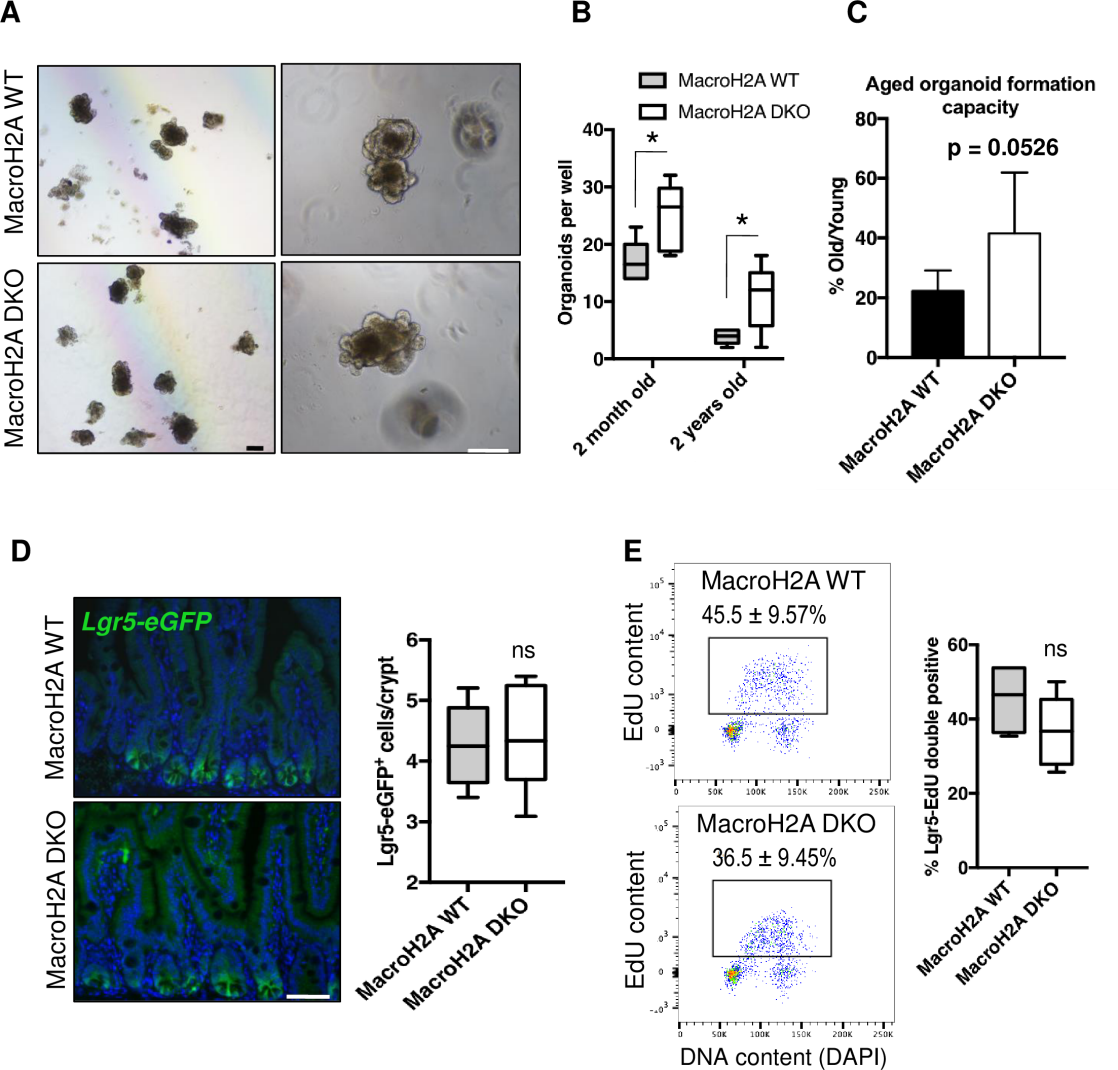
CBC frequency and activity in macroH2A DKO intestine. (A) Representative phase contrast images of macroH2A WT and DKO crypt-derived organoids, 7 days into culture. Left: 4x objective. Right: 10x objective. (B) Average resulting organoids per well (24-well tissue culture plate) from 100 crypts from macroH2A WT or DKO proximal jejunum from 2-month or 2-year old mice. N = 6 mice per condition, medians, quartiles and ranges of values shown. (C) Aged organoid formation capacity as defined by the average number of organoids that formed as a percent of the number of corresponding organoids that formed from 2-month old crypts per genotype. 10x objective. (D) Left: representative anti-eGFP immunofluorescence of macroH2A WT and DKO jejunum counterstained with DAPI (blue). Right: average Lgr5-eGFP^+^ cells per crypt. N = 6 mice per condition, medians, quartiles and ranges of values shown. (E) Left: representative flow cytometry plots of EdU content vs. DAPI of within *Lgr5-eGFP*^*+*^ subpopulations of macroH2A WT and DKO proximal jejunal crypt cells. Right: quantitation of *Lgr5-eGFP*/EdU double positivity as defined by boxed subpopulation on left. N = 4 mice per condition, medians, quartiles and ranges of values shown. *p<0.05, ns = not significant, Student’s *t*-test. Scale bars = 100μm.

In order to assess macroH2A DKO intestinal stem cell functionality, we isolated whole intestinal crypts from DKO and WT mice for *in vitro* organoid formation assays. Organoid growth is driven by ISCs, and both active CBCs and reserve ISCs are capable of initiating organoid formation.[11, 59] Phenotypically normal organoids were robustly generated from macroH2A DKO crypts (Fig 3A) at a strikingly greater frequency than macroH2A WT crypts (Fig 3B), suggesting that macroH2A DKO crypts may harbor more ISCs per crypt that are able to contribute to organoid genesis. This result was reproduced in crypts isolated from 2-year old macroH2A DKO and WT mice (Fig 3B). Since 2-year old macroH2A DKO crypts retained roughly equal organoid formation capacity compared to WT (Fig 3C), this suggests that macroH2A absence doesn’t affect the degree of intestinal stem cell exhaustion during aging.

We next sought to determine whether macroH2A DKO mice have different numbers of CBCs. To this end we bred macroH2A DKO and strain-matched WT mice into the *Lgr5-eGFP-IRES-CreERT2* reporter strain. Surprisingly, macroH2A DKO crypts contained equal numbers of CBCs per crypt as WT (Fig 3D) with functionally identical cell cycle profiles (Fig 3E). These data suggest that the increased DKO organoid formation was neither due to increased CBC numbers nor increased CBC proliferation.

### Reserve ISC frequency and activity in macroH2A DKO intestine

**Fig 4:**
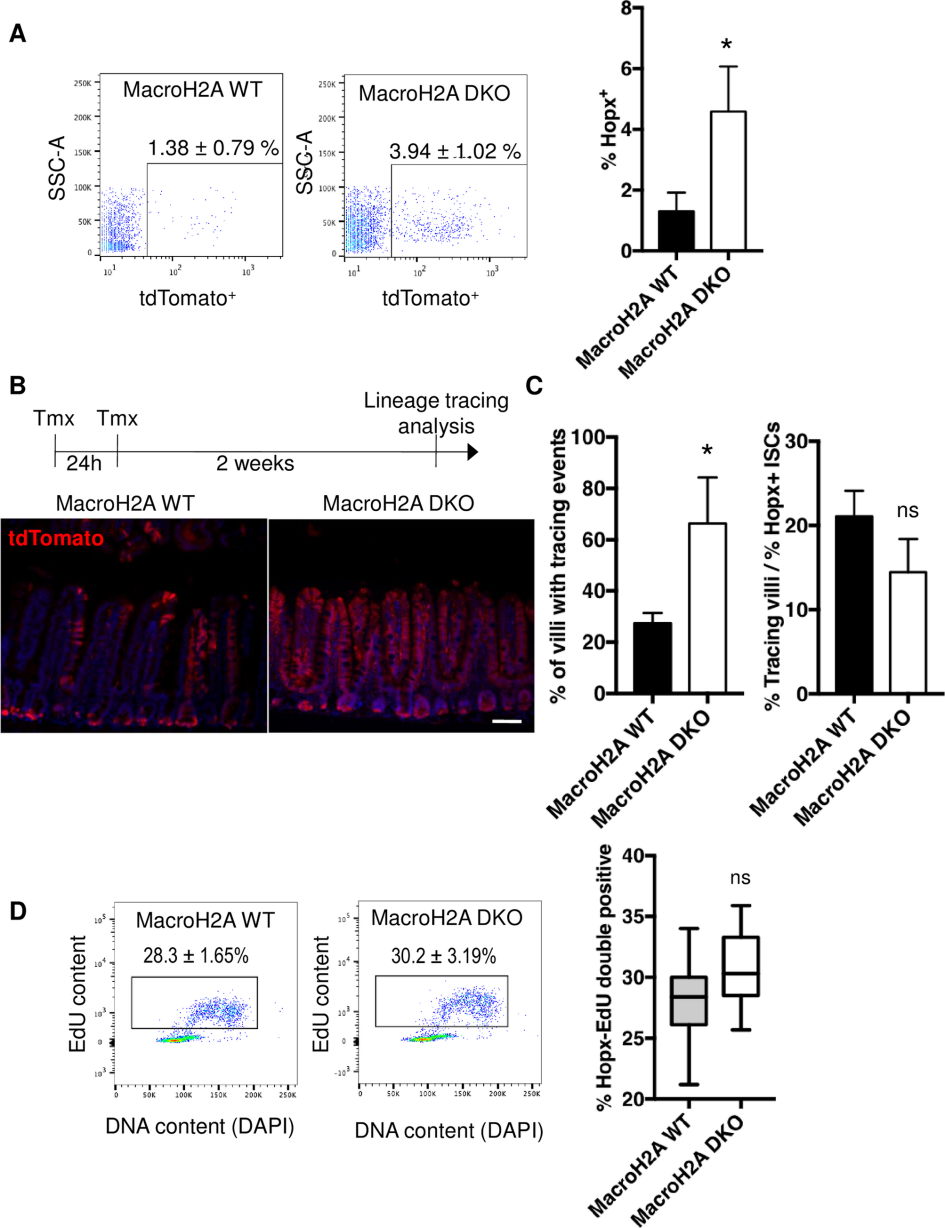
Reserve ISC frequency and activity in macroH2A DKO intestine. (A) Left: representative flow cytometry plots of SSC-A vs. *Hopx-tdTomato*^*+*^ signal in proximal small intestine crypt cells from macroH2A WT or DKO mice. Right: quantitation of *Hopx-tdTomato*^*+*^ population as a percentage of crypt epithelial cells. N = 5 mice per condition, mean ± SD. (B) Top: homeostatic lineage-tracing scheme: macroH2A WT and DKO *Hopx-CreERT2*::*Rosa26-LSL-tdTomato* mice were injected with 2mg tamoxifen for 2 consecutive days followed by a 2-week chase. Bottom: representative anti-tdTomato immunofluorescence (red) counterstained with DAPI (blue) of macroH2A WT and DKO proximal jejunum 2-weeks after induction of *Hopx-tdTomato* lineage tracing. 4x objective. (C) Left: quantitation of percentage of villi with tracing events after 2 week chase, N = 3 mice per condition, mean ± SD. Right: percentage of villi with tracing events normalized to percentage of *Hopx-tdTomato*^*+*^ ISCs during homeostasis (values in Fig 4A). N = 3 mice per condition, mean ± SD. (D) Left: representative flow cytometry plots of EdU content vs. DAPI of within *Hopx-tdTomato*^*+*^ subpopulations of macroH2A WT and DKO proximal jejunal crypt cells. Right: quantitation of *Hopx-tdTomato*/EdU double positivity as defined by boxed subpopulation on left. N = 7 mice per condition, medians, quartiles and ranges of values shown. *p<0.05, ns = not significant, Student’s *t*-test. Scale bar = 100μm.

To interrogate the reserve ISC compartment in mice without macroH2A, we bred macroH2A DKO and strain-matched WT mice into the *Hopx-Cre-ERT2*::*Rosa26-LSL-tdTomato* reporter strain.[9, 10] Remarkably, macroH2A DKO crypts contained significantly more putative Hopx-CreER^+^ reserve ISCs than WT (Fig 4A), suggesting presence of macroH2A within reserve ISCs or the ISC niche may limit reserve ISC numbers. MacroH2A DKO reserve ISCs also exhibited significantly greater steady-state lineage tracing compared to WT reserve ISCs (Fig 4B and 4C). However, this increased tracing could not be attributed to increased reserve ISC cycling, as no statistically significant increase in EdU incorporation was observed within macroH2A DKO reserve ISCs (Fig 4D). Rather, the increased tracing appeared to be largely a reflection of the increased size of the reserve ISC pool, as normalization of tracing events to reserve ISC cell numbers revealed no significant difference between macroH2A DKO and WT cohorts (Fig 4C). In sum, these results reveal that while macroH2A DKO reserve ISCs are almost 3 times as abundant as WT, they are not significantly more proliferative than WT.

### Regeneration and DNA damage response in macroH2A DKO intestine

**Fig 5:**
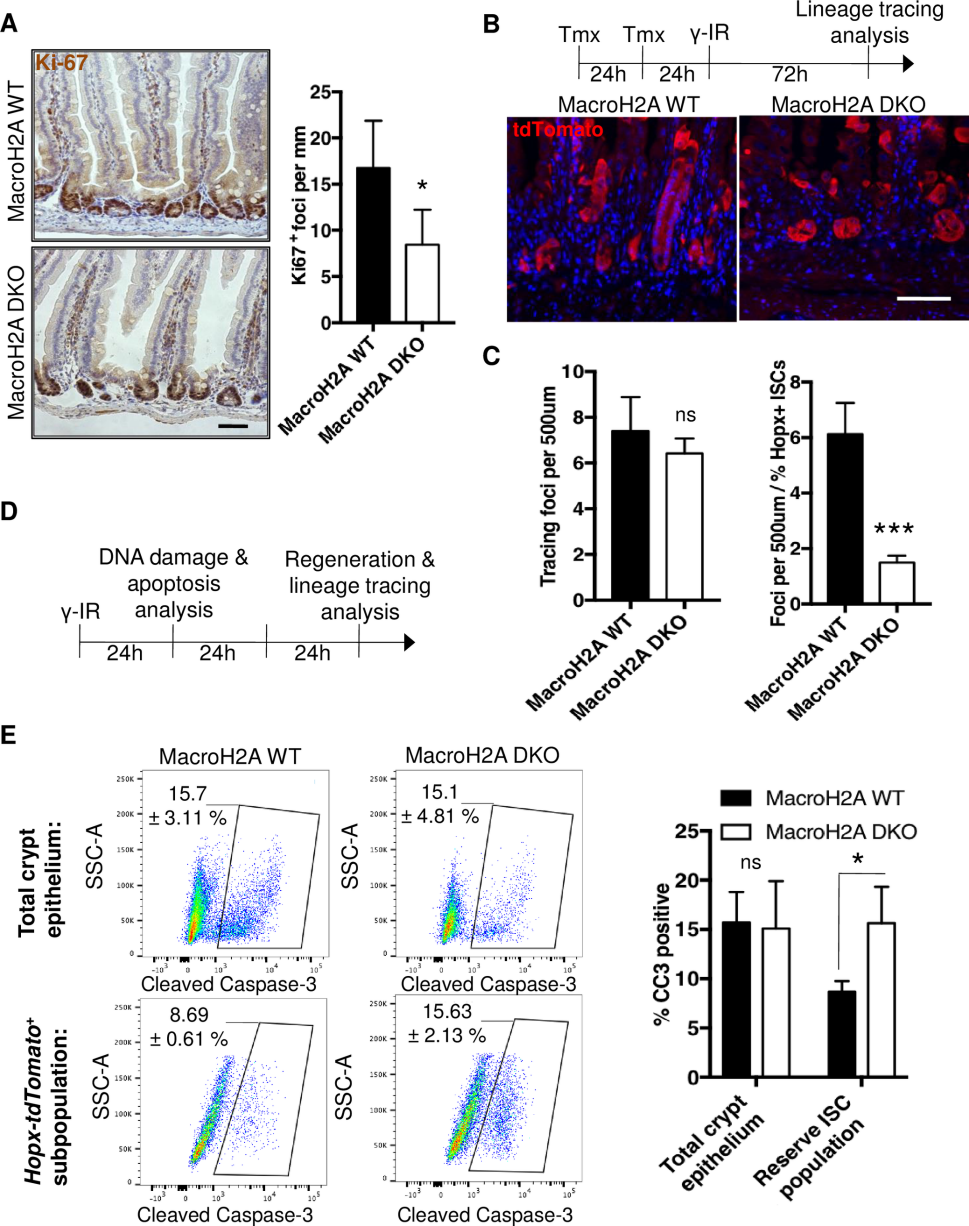
Regeneration and DNA damage response in macroH2A DKO intestine. (A) Left: representative images of Ki-67 immmunohistochemistry within macroH2A WT and DKO proximal jejunum 3 days after exposure of mice to 12 Gy whole body γ-irradiation. 10x objective. Right: quantitation of Ki67^+^ nascent crypt foci per mm. N = 3 mice per condition, mean ± SD. (B) Top: post-IR lineage tracing scheme: macroH2A WT or DKO *Hopx-CreERT2*::*Rosa26-LSL-tdTomato* mice were injected with 2mg tamoxifen 48h and 24h prior to treatment with 12 Gy whole-body gamma irradiation, and 72h later sacrificed for analysis. Bottom: representative immunofluorescence of tdTomato lineage tracing (red) counterstained with DAPI (blue) within macroH2A WT and DKO crypts 72 hours after γ-irradiation. 30x objective (C) Left: quantitation of tdTomato tracing events per 500μm, N = 3 mice per condition, mean ± SD. Right: quantitation of tdTomato tracing events per 500μm normalized to percentage of *Hopx-tdTomato*^*+*^ ISCs during homeostasis (values in Fig 4A), N = 3 mice per condition, mean ± SD. (D) Experimental scheme highlighting the timing of DNA damage and apoptosis analysis (24h post IR) and regeneration and lineage tracing analysis (72h post IR) (E) Left: flow cytometry plots of SSC-A vs. cleaved caspase-3 content within total crypt epithelium or *Hopx-tdTomato*^*+*^ subpopulations of macroH2A WT and DKO proximal jejunal crypt cells 24 hours after γ-irradiation. Right: quantitation of total crypt epithelium CC3 positivity and *Hopx-tdTomato*^+^/CC3 double positivity as defined by boxed subpopulation on left. N = 3 mice per condition, mean ± SD. *p<0.05, ***p<0.0005, ns = not significant, Student’s *t*-test. Scale bars = 100μm.

Reserve ISCs are known to be resistant to DNA damage and required for epithelial regeneration following exposure to high-dose γ-radiation that quantitatively ablates actively cycling cells including CBCs.[4, 6, 7, 12, 15, 16] To test the contribution of macroH2A DKO reserve ISCs to intestinal regeneration following injury, we subjected macroH2A WT and DKO mice to high-dose (12Gy) γ-radiation. Strikingly, macroH2A DKO intestine exhibited an impaired regenerative response compared to WT with significantly fewer nascent regenerative crypt foci per millimeter forming after irradiation (Fig 5A). Interestingly, irradiation of mice two days after Hopx-CreER^+^ lineage tracing initiation revealed comparable numbers of clonal tracing events in regenerative crypts between macroH2A DKO and WT (Fig 5B and 5C). This observation reveals a significant decrease in tracing from macroH2A DKO reserve ISCs versus WT on a per-cell basis (Fig 5C), and suggests that macroH2A DKO reserve ISCs have increased DNA damage sensitivity. To test this, we assayed macroH2A DKO intestine for DNA damage and apoptosis prior to regeneration at an earlier time point–one day after irradiation (Fig 5D). Somewhat paradoxically, macroH2A DKO and WT intestine neither showed a significant difference in crypt apoptosis at large nor DNA damage signal clearance in the crypt 1 day after irradiation (S2 Fig). This is perhaps not surprising, as macroH2A was shown to neither affect H2AX phosphorylation nor γ-H2AX signal clearance *in vitro*.[53] However, the reserve ISC compartment of macroH2A DKO crypts exhibited a higher incidence of cleaved caspase-3 immunoreactivity (Fig 5E), indicating that macroH2A DKO reserve ISCs disproportionately undergo apoptosis and are thus aberrantly radiosensitive. Importantly, macroH2A DKO crypt epithelium at large was not significantly more apoptotic than WT (Fig 5E), corroborating our previous results (S2 Fig). Taken together, these data suggest that macroH2A bestows reserve ISCs with resistance to radiation-induced DNA damage.

### Influence of macroH2A on intestinal tumorigenesis

Colorectal cancer (CRC) progression is directly correlated with an increase in the expression of an ISC transcriptional signature, and both Wnt^High^ CBCs and Wnt^Negative^ radioresistant cells have been implicated as potential cells-of-origin in colorectal tumorigenesis.[7, 60–62] Our findings thus far indicate that macroH2A DKO crypts exhibit increased ISC activity in organoid formation assays (Fig 3A–3C), increased reserve ISC numbers (Fig 4A), and reduced reserve ISC DNA damage tolerance (Fig 5E). Given that macroH2A has been implicated as a tumor suppressor in several cancers including CRC,[54–58] we asked whether macroH2A absence might influence intestinal tumorigenesis.

**Fig 6:**
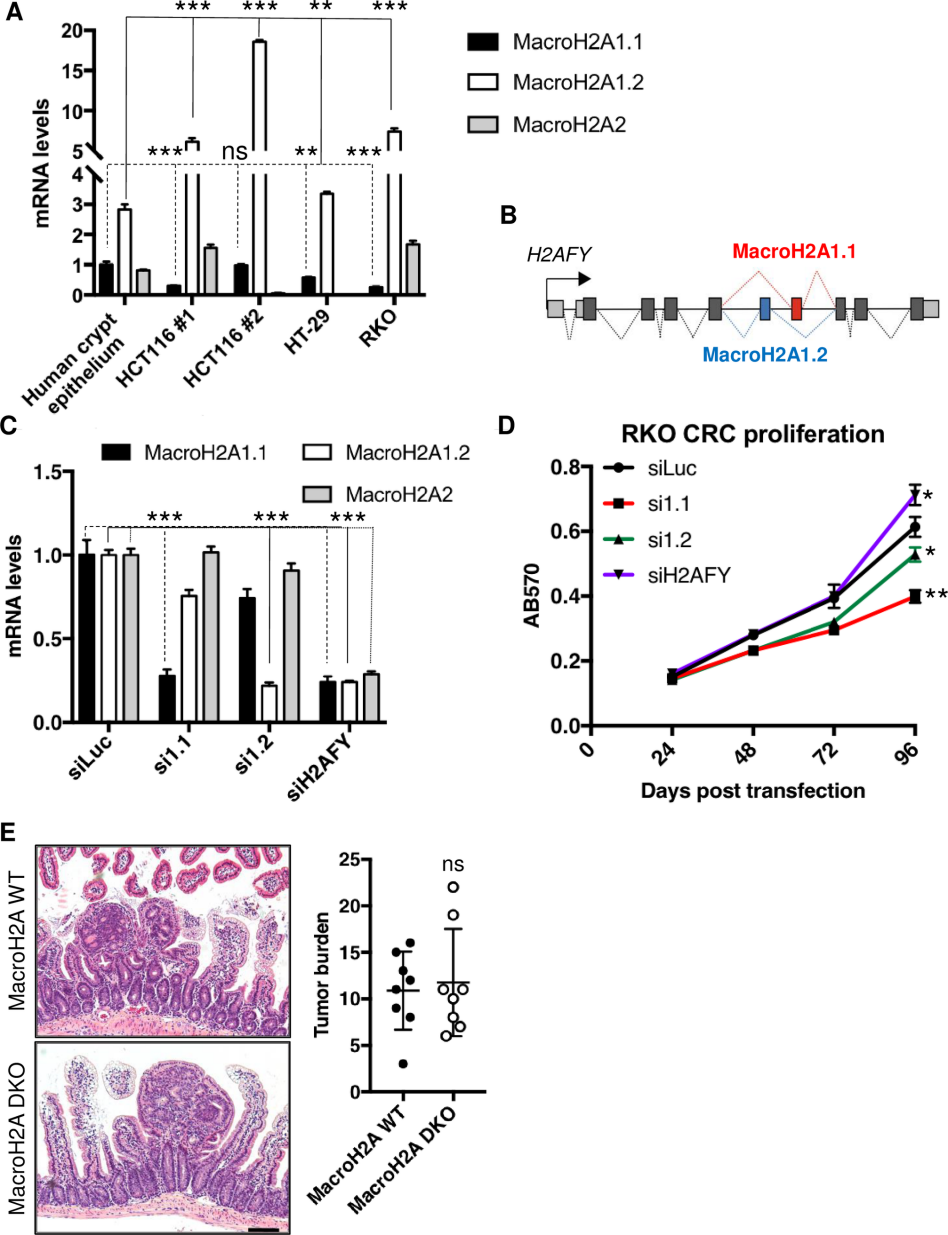
MacroH2A’s influence of intestinal tumorigenesis. (A) MacroH2A mRNA level analysis of healthy human intestinal crypt epithelium and human CRC cell lines. ΔΔCT method, values normalized to *GAPD*. N = 3 per condition, mean ± SD. (B) Graphical depiction of the *H2AFY* gene and its exons, including the mutually-exclusive exons of the macroH2A1.1 and macroH2A1.2 splice variants. (C) MacroH2A siRNA knockdown validation in RKO CRC cell line. ΔΔCT method, values normalized to *GAPD* independently per macroH2A primer relative to luciferace knockdown control. N = 3 per condition, mean ± SD. (D) MTT cell proliferation assay of RKO cell line during macroH2A1.1, 1.2, *H2AFY*, or control luciferace RNAi knockdown. N = 3 per condition, mean ± SD. (E) Left: representative H&E images of macroH2A WT and DKO Apc^min^-derived tumors within the small intestine. 4x objective. Right: quantitation of average total tumors within entire small intestine of macroH2A WT and DKO. N = 8 mice per condition, mean ± SD. *p<0.05, **p<0.005, ***p<0.0005, ns = not significant, Student’s *t*-test. Scale bar = 100μm.

Consistent with a prior report,[55] we observed decreased macroH2A1.1 expression in several human CRC cell lines relative to healthy human intestinal crypt epithelium (Fig 6A). Concomitantly, the non-PAR binding macroH2A1.2 exhibited greater expression in several CRC lines, suggesting selection for increased macroH2A1.2 vs. macroH2A1.1 isoform splicing disparity in these cancers (Fig 6A). MacroH2A1.2 and macroH2A1.1 are produced by mutually exclusive exon inclusion spicing events (Fig 6B), therefore our data corroborate literature that suggests that the PAR-binding isoform macroH2A1.1 has tumor suppressive activity.[55–58]

To simulate the transcriptional environment of macroH2A DKO ISCs in human CRCs, we used RNAi to knock down macroH2A within two CRC lines that exhibited both a pronounced increase in macroH2A1.2 and a prominent decrease in macroH2A1.1. Surprisingly, knockdown of either macroH2A1.1 or macroH2A1.2 modestly but significantly reduced proliferation (Fig 6C and 6D, S3 Fig). While the siRNA knockdowns were robust and specific, particularly in RKOs (Fig 6C), we cannot rule out the possibility of altered macroH2A1 isoform genomic deposition following reciprocal splice variant depletion, and the functional consequences thereof. Interestingly, pan-*H2AFY* knockdown resulted in a modest increase in RKO and HCT116 CRC proliferation (Fig 6C and 6D, S3 Fig), suggesting that total macroH2A loss may increase CRC proliferation slightly and contribute subtly to oncogenesis.

Finally, to test the influence of macroH2A absence on intestinal tumorigenesis in a more physiological setting, we bred macroH2A DKO and WT mice into the *Apc*^*min/+*^ mouse model of intestinal transformation[63] and quantified adenoma formation. On average, macroH2A DKO mice did not develop more tumors compared to WT (Fig 6E), indicating that macroH2A absence does not hypersensitize the intestinal epithelium to oncogenic stress caused by loss of heterozygosity in the *Apc*^min/+^ model. Further, macroH2A DKO adenomas were not overtly more proliferative than their WT counterparts (S3 Fig), suggesting that macroH2A doesn’t robustly influence tumor initiation. These findings are consistent with prior work which observed no increase in spontaneous tumor formation in ageing macroH2A DKO mice.[19] Taken together, these data suggest that macroH2A has no significant tumor suppressive function in the intestinal epithelium with respect to adenoma initiation resulting from Apc loss, yet do not rule out the possibility that macroH2A content influences further tumor growth and behavior following establishment.

## Discussion

This study identified for the first time a role for the histone variant macroH2A in the function of somatic stem cells *in vivo*. In spite of the observed radiosensitivity within macroH2A DKO reserve ISCs, macroH2A is ostensibly dispensable during intestinal homeostasis. This is perhaps not surprising, as macroH2A DKO mice are ordinarily healthy, yet at the same time are described as smaller, more perinatal death-prone, and less vigorous overall than WT counterparts.[19] It is therefore interesting that macroH2A DKO mice are more sensitive to genotoxic γ-irradiation, as this is further evidence that macroH2A DKO mice are less robust.

As with our *in vivo* study, macroH2A perturbation alongside genotoxic stress has been of great consequence in a number of *in vitro* studies. In one example, simultaneous macroH2A knockdown and viral challenge increased the ‘transcriptional noise’ of many genes.[51] In another study, macroH2A1.1 and PARP-1 were shown to coordinate proper hsp70 expression following heat-shock induction.[38] Further, two notable studies highlight roles for both macroH2A1.1 and macroH2A1.2 in directing DNA damage response (DDR) element localization following targeted double strand break (DSB) induction. PAR-binding macroH2A1.1 knockdown was shown to impair PARP-1 recruitment to DSB sites, a key early step in the DDR.[53] Additionally, knockdown of non-PAR-binding macroH2A1.2 significantly reduced BRCA1 recruitment to break sites and in turn reduced DSB resolution via homology-directed repair (HDR).[52]

Based on the literature and our study’s observed increase in cleaved caspase-3 staining within macroH2A DKO reserve ISCs compared to WT, it’s tempting to speculate that macroH2A DKO reserve ISCs are less effective at DNA repair than WT, and thus excessively undergo apoptosis after suffering DNA damage. Specific DDR deficiencies within macroH2A DKO reserve ISCs remain unknown, but possibilities include reduced Chk2 kinase phosphorylation, a DDR signaling hallmark shown to be disrupted upon macroH2A knockdown.[50] Another possibility is that macroH2A DKO reserve ISCs are less able to recruit BRCA1 to DSB sites and thus disproportionately undergo non-homologous end joining rather than the less error-prone HDR.[52] Further studies are needed to determine which DDR deficiencies macroH2A DKO reserve ISCs may suffer from.

In our study, we discovered that macroH2A DKO intestine has almost 3 times as many reserve ISCs than WT under steady-state conditions. This result is perhaps not surprising as it’s been shown that macroH2A knockdown can increase somatic stem cell self-renewal *in vitro*.[49] Interestingly, macroH2A DKO reserve ISCs are not significantly more proliferative than WT. This could suggest that more DKO reserve ISCs are established early in development, or alternatively that DKO reserve ISCs undergo more frequent self-renewal versus commitment divisions. Additionally, we cannot rule out the possibility of non cell-autonomous influences on ISC numbers, including from the macroH2A DKO ISC niche. Future experiments aimed at understanding macroH2A’s role in ISC development and specification are needed to further characterize the macroH2A DKO reserve ISC.

Our research has shed light on macroH2A’s purported tumor suppressive role. Since macroH2A has been shown to provide functional robustness against genotoxic stress in several studies[38, 50–53] including our own, it follows that macroH2A may also insulate against oncogenesis, at least in part by bolstering DNA repair. It is therefore interesting that macroH2A DKO in an *Apc*^*min/+*^ background does not result in increased tumorogenesis relative to WT, yet this result is in agreement with the observation that macroH2A DKO mice are not more susceptible to spontaneous cancer.[19] Another nuance to the study of macroH2A in cancer is that macroH2A1.1 and macroH2A1.2 may have distinct influences on oncogenesis. MacroH2A1.1 has more often than macroH2A1.2 been described as a bona-fide tumor suppressor.[55–58] Interestingly, another study found that macroH2A1 can potentiate silencing, heterochromatin formation, and hypermethylation of the tumor suppressor p16 in CRC, but the work did not distinguish between macroH2A1.1 and macroH2A1.2.[64] These insights highlight the importance of developing tools that distinguish between the individual effects of macroH2A isoforms, particularly the macroH2A1 splice variants–both in terms of variant expression as well as subgenomic localization. Understanding the individual roles of the macroH2A isoforms will indeed prove critical to further characterizing the role of macroH2A in cancer, in ISCs, and undoubtedly in other adult stem cell systems as well.

## Supporting information

S1 Fig Differentiated intestinal epithelial cell quantitation. (A) Quantitation of Lysozyme C^+^ Paneth cells per crypt, N = 3 per condition, mean ± SD. (B) Quantitation of chromogranin A^+^ enteroendocrine cells per villus, N = 3 per condition, mean ± SD. (C) Quantitation of Alcian Blue stained goblet cells per 500 microns, N = 3 per condition, mean ± SD. *p<0.05, ns = not significant, Student’s *t*-test.
(TIF)

S2 Fig γ-H2AX and CC3 foci quantitation after γ-irradiation. (A) Left: representative γH2AX immunofluorescence (green) counterstained with DAPI (blue) within macroH2A WT and DKO proximal small intestine 24 hours after exposure to 12 Gy. 10x objective. Middle: quantitation of percent of crypts with γH2AX signal during homeostasis or 24 hours after 12Gy. Right: quantitation of average γH2AX cells per crypt with at least one CC3^+^ cell 24 hours after γ-irradiation. N = 3 mice per condition, mean ± SD. (B) Left: representative images of cleaved-caspase 3 (CC3) immunohistochemistry within macroH2A WT and DKO proximal small intestine 24 hours after exposure to 12 Gy. 40x objective. Middle: quantitation of percent of crypts with CC3 signal during homeostasis or 24 hours after 12Gy. Right: Quantitation of average CC3^+^ cells per crypt with at least one CC3^+^ cell 24 hours after γ-irradiation. N = 3 mice per condition, mean ± SD. Scale bar = 100μm. ns = not significant, Student’s *t*-test.
(TIF)

S3 Fig Effects of macroH2A knockdown on HCT116 growth. (A) MacroH2A siRNA knockdown validation in HCT116 CRC cell line. ΔΔCT method, values normalized to *GAPD* independently per macroH2A primer relative to luciferace knockdown control. N = 3 per condition, mean ± SD. (B) MTT cell proliferation assay of HCT116 cell line during macroH2A1.1, 1.2, *H2AFY*, or control luciferace RNAi knockdown. N = 3 per condition, mean ± SD. (C) Representative Ki67 immunofluorescence of macroH2A WT and DKO proximal small intestine adenoma tissue. *p<0.05, **p<0.005, ***p<0.0005, ns = not significant, Student’s *t*-test.
(TIF)
