# Supplementary figures and images for: The histone variant macroH2A confers functional robustness to the intestinal stem cell compartment
Source: PLoS One. 2017 Sep 21;12(9):e0185196. doi: 10.1371/journal.pone.0185196 (PMC5608326; doi:10.1371/journal.pone.0185196)

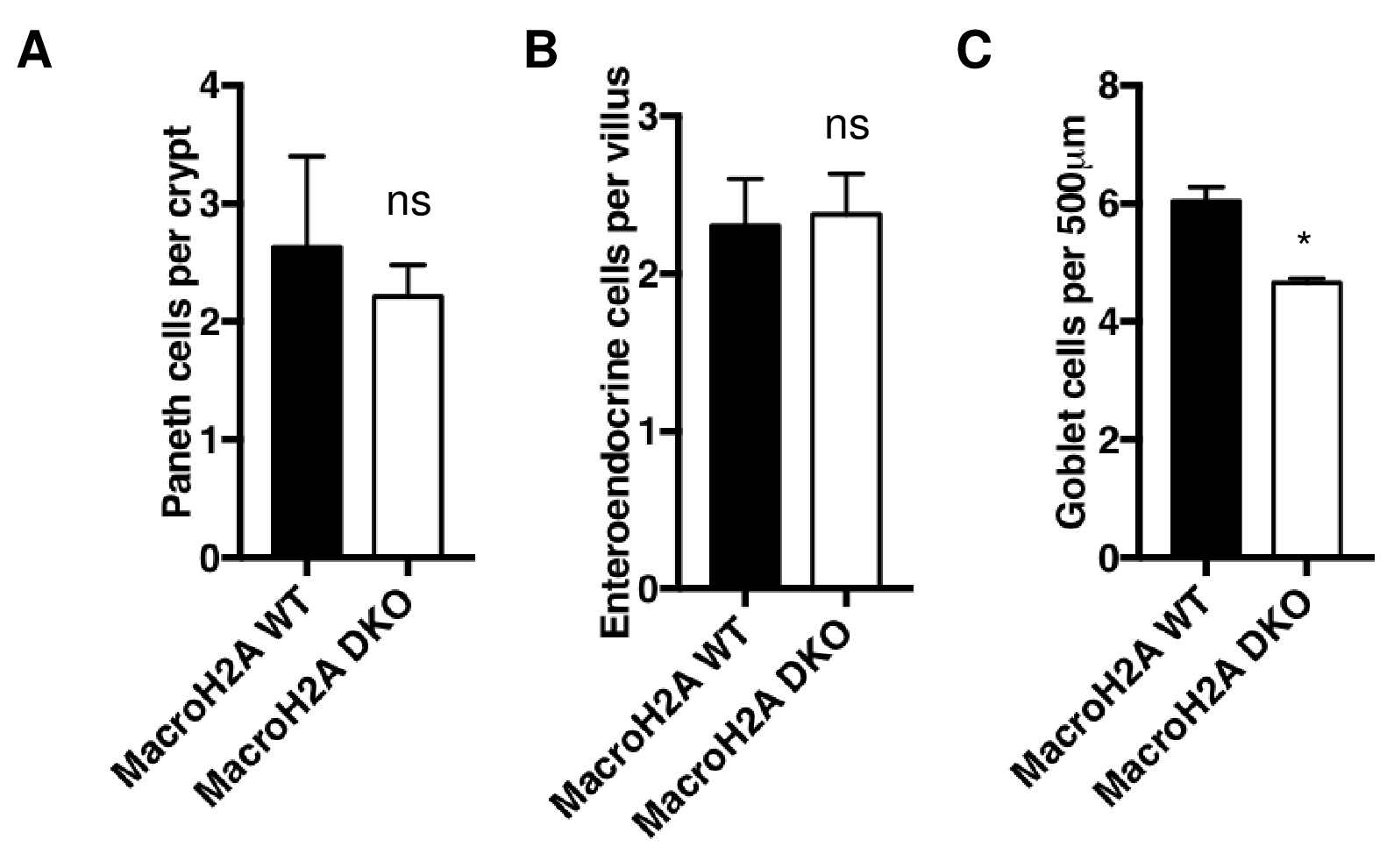

Supplement: S1 Fig — (A) Quantitation of Lysozyme C+ Paneth cells per crypt, N = 3 per condition, mean ± SD. (B) Quantitation of chromogranin A+ enteroendocrine cells per villus, N = 3 per condition, mean ± SD. (C) Quantitation of Alcian Blue stained goblet cells per 500 microns, N = 3 per condition, mean ± SD. *p<0.05, ns = not significant, Student’s t-test. (TIF) [file pone.0185196.s001.tif]

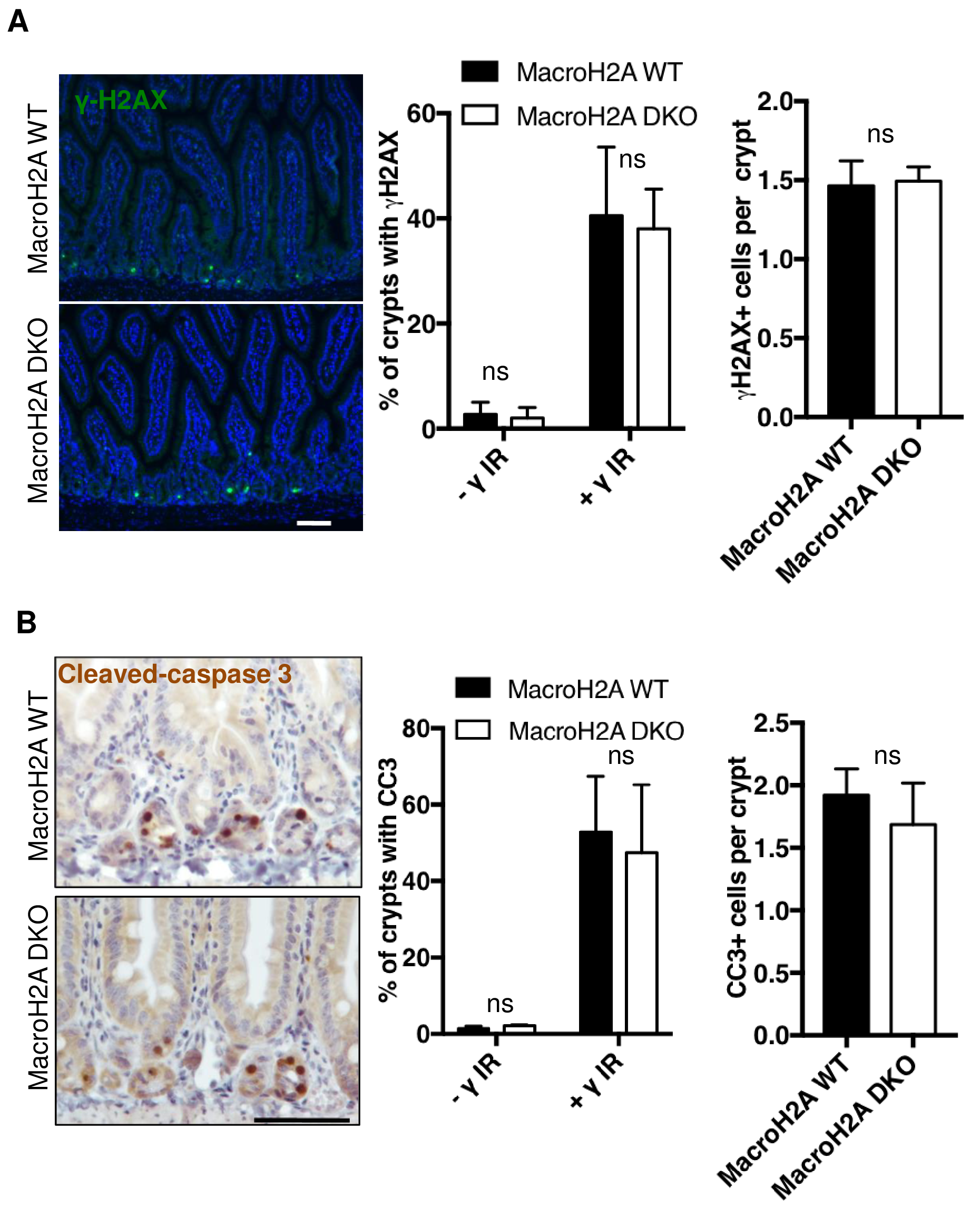

Supplement: S2 Fig — (A) Left: representative γH2AX immunofluorescence (green) counterstained with DAPI (blue) within macroH2A WT and DKO proximal small intestine 24 hours after exposure to 12 Gy. 10x objective. Middle: quantitation of percent of crypts with γH2AX signal during homeostasis or 24 hours after 12Gy. Right: quantitation of average γH2AX cells per crypt with at least one CC3+ cell 24 hours after γ-irradiation. N = 3 mice per condition, mean ± SD. (B) Left: representative images of cleaved-caspase 3 (CC3) immunohistochemistry within macroH2A WT and DKO proximal small intestine 24 hours after exposure to 12 Gy. 40x objective. Middle: quantitation of percent of crypts with CC3 signal during homeostasis or 24 hours after 12Gy. Right: Quantitation of average CC3+ cells per crypt with at least one CC3+ cell 24 hours after γ-irradiation. N = 3 mice per condition, mean ± SD. Scale bar = 100μm. ns = not significant, Student’s t-test. (TIF) [file pone.0185196.s002.tif]

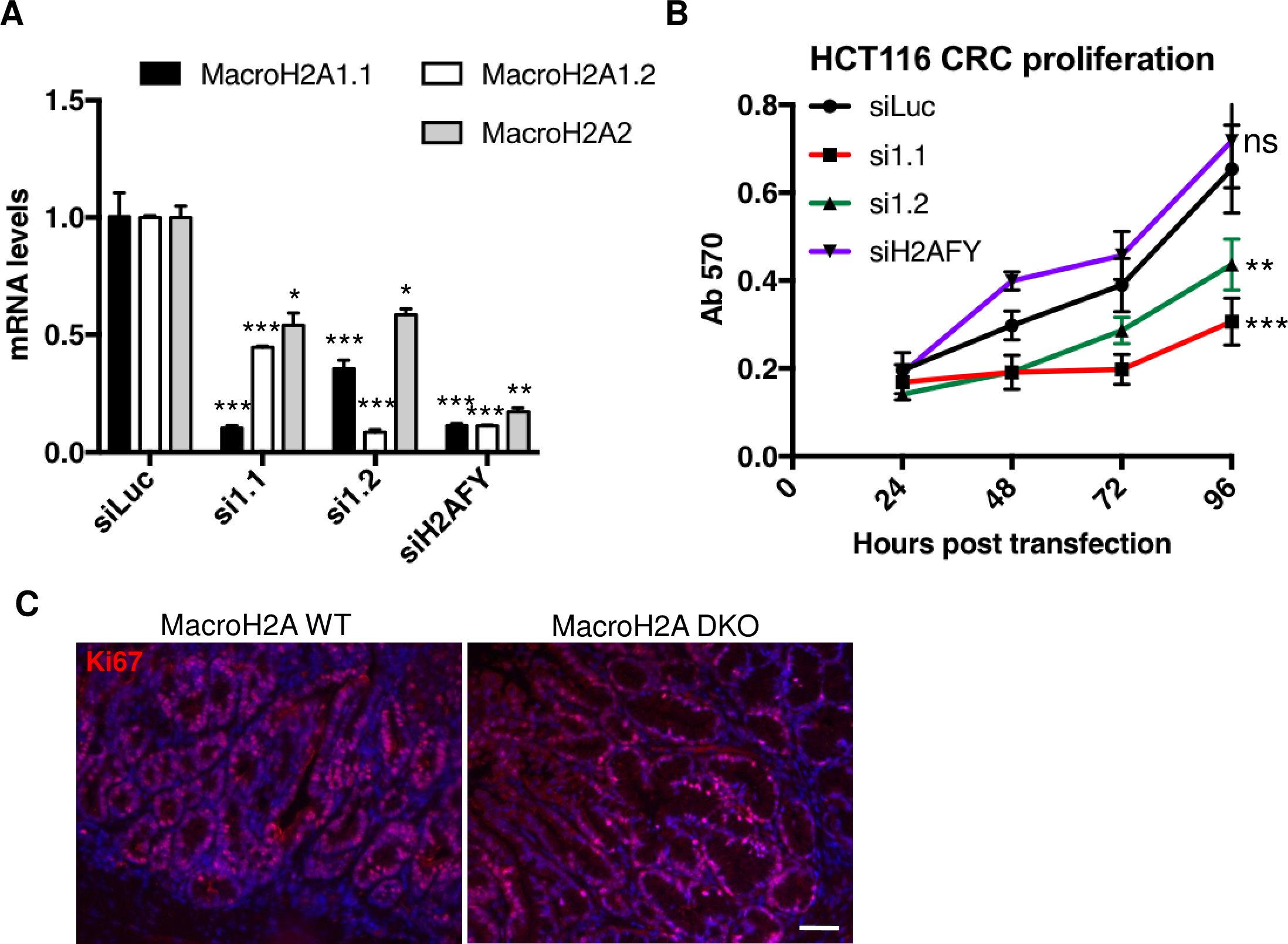

Supplement: S3 Fig — (A) MacroH2A siRNA knockdown validation in HCT116 CRC cell line. ΔΔCT method, values normalized to GAPD independently per macroH2A primer relative to luciferace knockdown control. N = 3 per condition, mean ± SD. (B) MTT cell proliferation assay of HCT116 cell line during macroH2A1.1, 1.2, H2AFY, or control luciferace RNAi knockdown. N = 3 per condition, mean ± SD. (C) Representative Ki67 immunofluorescence of macroH2A WT and DKO proximal small intestine adenoma tissue. *p<0.05, **p<0.005, ***p<0.0005, ns = not significant, Student’s t-test. (TIF) [file pone.0185196.s003.tif]
